# A fast emergency department triage score based on mobility, mental status and oxygen saturation compared with the emergency severity index: a prospective cohort study

**DOI:** 10.1093/qjmed/hcad160

**Published:** 2023-07-03

**Authors:** H B Riedel, T Espejo, R Bingisser, J Kellett, C H Nickel

**Affiliations:** Emergency Department, University Hospital Basel, Petersgraben 2, 4031 Basel, Schweiz; Emergency Department, University Hospital Basel, Petersgraben 2, 4031 Basel, Schweiz; Emergency Department, University Hospital Basel, Petersgraben 2, 4031 Basel, Schweiz; Department of Emergency Medicine, Odense University Hospital, J. B. Winsløws Vej 4, 5000 Odense, Denmark; Emergency Department, University Hospital Basel, Petersgraben 2, 4031 Basel, Schweiz

## Abstract

**Background:**

Waiting for triage in overburdened emergency departments (ED) has become an increasing problem, which endangers patients. A fast triage system to rapidly identify low-acuity patients should divert care and resources to more urgent cases.

**Aim:**

The objective of this study was to compare the performance of the Kitovu Hospital fast triage (KFT) score with the Emergency Severity Index (ESI), using mortality and hospital admission as proxies for the patients’ acuity.

**Design:**

This is a prospective observational study of consecutive patients presenting to a Swiss academic ED.

**Methods:**

Patients were prospectively triaged into one of five ESI strata and retrospectively assessed by the KFT score, which awards one point each for altered mental status, impaired mobility and oxygen saturation <94%.

**Results:**

The KFT score had a lower discrimination than the ESI for hospital admission, but a higher discrimination for mortality from 24 h to 1 year after ED presentation. A total of 5544 (67%) patients were assigned to the lowest acuity by the KFT score compared with 2374 (28.7%) by the ESI; there was no significant difference in the 24-h mortality of patients who were deemed low acuity by either score.

**Conclusion:**

Compared to the ESI, the KFT score identifies more than twice as many patients at low risk of early death. Therefore, this score might help to identify patients who could be managed through alternative pathways. This may be particularly helpful in situations of ED crowding and access block.

## Background

Waiting for triage has been identified as a threat to safety in the emergency department (ED).^1^ The intended purpose of triage can be undermined by long lines of patients queuing up to be seen, which inevitably hinders essential time-critical treatment. Even under routine circumstances, the time from ED arrival to triage can take longer than 10 min in more than half of all high-acuity patients; during busier times, this critical time span increases, so that most high-acuity patients may not be seen within the recommended time period.^1^ In a recent policy statement, the American College of Emergency Physicians and the Emergency Nurses Association stated that extended triage questioning also creates a preventable delay in caring for patients and should not interfere with timely access to needed care.^2^ Furthermore, limiting the extent of initial triage might be advantageous.^3^^,^^4^

Preventable early death after ED discharge must also be avoided.^5^ Therefore, several prognostic factors to predict short-term mortality have been studied. Mental status,^6^ impaired mobility,^7^ and vital signs^5^ are associated with early mortality. Not surprisingly, all three parameters are included in scores with good prognostic power for short-term mortality.^8^ Patients with normal vital signs and mobility showed both low early^8^^,^^9^ and low 1-year mortality.^9^ Oxygen saturation is one of the most valuable vital signs for triage.^10^ In a busy ED, all these parameters may rapidly identify a patient at low risk of early death, who could be managed as outpatient. ‘Red flag’ situations in which patients should not wait are rapidly and easily identified by the Emergency Severity Index (ESI) algorithm and other commonly used triage systems.^11^^,^^12^ These include presentations such as confusion, lethargy and disorientation as well as severe pain and distress.^13^ Furthermore, improving the outflow of patients is also important, as it is a major driver of ED crowding^14^; efficient triage should not only rapidly identify those in need of urgent care, but also patients who can be safely discharged and managed elsewhere. This reverse triage^15^ may be particularly helpful in situations of ED crowding and access block.^16^

The purpose of triage is to stratify patients according to their need to be timely managed (i.e. their acuity). There is no gold standard metric of acuity; therefore, most triage validation studies have used predictive validity based on resources, such as ICU and hospital admission, or early mortality.^11^^,^^17^^,^^18^ Prognostic information, such as long-term mortality, may also support appropriate and prudent disposition decisions.^8^ In Kitovu Hospital, Uganda, a simple, fast triage [Kitovu Hospital fast triage (KFT)] score, based on mobility, mental status and oxygen saturation, was developed and locally validated against 24 h mortality and the need for hospital admission.^19^ We hypothesized that the KFT score might be useful in any ED when demand overwhelms the available resources. The aim of this prospective observational study was to compare predictive validity of the KFT score with the ESI.

## Methods

### Study design

Secondary analysis of a prospective observational study designed to improve triage in the University Hospital Basel, Switzerland, a tertiary hospital with over 52 000 ED visits per year. All consecutively presenting patients from 30 January 2017 to 20 February 2017 and from 18 March 2019 to 20 May 2019 were included, 24 h a day, 7 days a week.

### Ethics

The study protocol was approved by a local ethics committee (Project ID: 236/13; http://eknz.ch; NCT03892551). Written informed consent was not required in this study, only patients who proactively refused were excluded. The study was conducted according to the STROBE guidelines.

### Selection of participants

During the study period, all consecutive patients presenting to the ED were eligible. Obstetric, ophthalmic and paediatric patients were not included, as they were directed to nearby specialist centres for treatment. Patients denying consent were excluded. Patients unable to communicate due to severe dementia, intoxications, delirium, coma, or insurmountable language barriers were not part of the analysis. Patients who represented during the study period were also excluded from this secondary analysis.

### Data collection

Upon presentation to the ED, a member of the study team screened and interviewed every patient, documented their presenting symptoms and registered their data in the electronic health record (EHR). All patients presenting 24 h a day and 7 days a week were included. The study team consisted of trained medical students.

All data were recorded on machine-readable case report forms, which were subsequently scanned, and the data were then cleaned in a two-step process. First, by ED administrators for handwriting issues and then by an external company, Swiss Post^®^, which was also responsible for the transfer of data into the database. All other data from the patients’ EHR were also transferred into the database, using the unique patient ID to match it with the EHR.

### Triage process

All patients presenting to the ED were routinely assessed by ESI, a five-level triage algorithm used to stratify patients regarding acuity and resources. ESI level 1 is the highest acuity, indicating the need of immediate life-saving interventions; ESI level 2 indicates a high-risk situation or abnormal vital signs. Patients with normal vital signs requiring more than one resource are assigned to ESI level 3, those in need of one resource to level 4 and those without need of external resources are ESI level 5, the least urgent level.^17^

A complete set of vital signs was obtained in all patients and their KFT score was determined by awarding one point each for altered mental status, impaired mobility and oxygen saturation <94%.^19^ Altered mental status was defined by the ‘Alert, Voice responsive, Pain responsive and Unresponsive (AVPU) metric’; in alert patients, no KFT points were attributed, and in all others, 1 point was attributed. Normal mobility was defined as the ability to walk to the treatment room without any help (e.g. crutches, or wheelchairs). All Patients unable to walk without help received one point in the KFT score. Oxygen saturation was measured by the triage staff during triage using a pulse-oximetric sensor.

### Missing data

Patients with missing data for any of the three metrics used in our analysis were excluded.

### Outcomes

The primary outcome of the study was to determine construct validity of the KFT score using short-term survival (i.e. 24-h mortality).

The secondary outcome was to determine construct validity of the KFT score for the use of resources (i.e. admission) and long-term prognosis (i.e. 1-year mortality).

### Statistical analysis

For descriptive statistics, categorical variables are shown in counts and frequencies, metric variables are presented as mean with standard deviation (SD). Variable comparisons for patients with missing data for KFT calculation (saturation, gait, mental status) was tested using Kruskal–Wallis test, Chi-square test or exact Fisher test when appropriate.

Receiver operating characteristic (ROC) curves demonstrating discrimination of KFT and ESI for admission, 24-h, 7-day and 1-year mortality were calculated by area under the curve (AUC) according to the method of Hanley and McNeil.^20^ The difference of ROC curves was calculated by bootstrapping.

The multivariate logistic regression analysis models were corrected for age and gender. They were used to calculate odds ratios (OR), 95% confidence intervals (CIs) and *P*-values. Patients with ESI 3 and KFT 0 were considered the reference group. *P*-values <0.05 were defined as significant. Kaplan–Meier curves for 30-day survival compared by KFT score were compared using the log-rank test. All analyses were performed using the R Studios software (Version 4.2.1).^21^

## Results

### Main results

**Figure 1.**
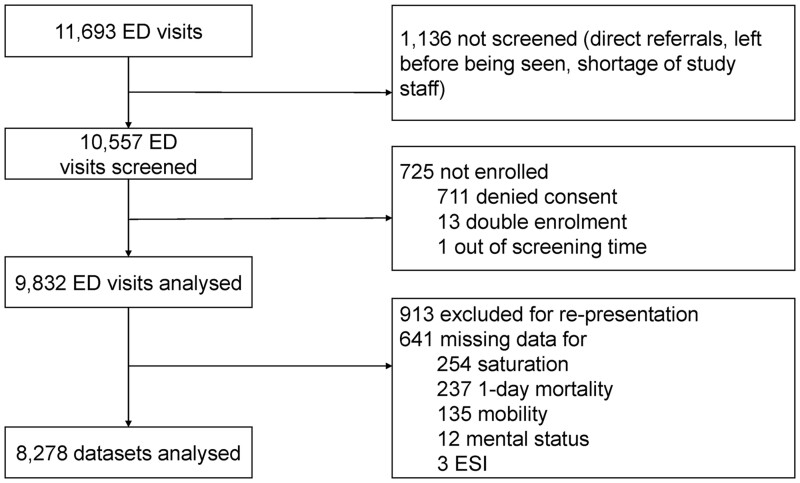
Patient selection process. All consecutive patients presenting to the ED providing informed consent were included. Patients were excluded if they were represented during the study time or had missing data for saturation, mobility, mental status, ESI or 1-day mortality.

**Figure 2.**
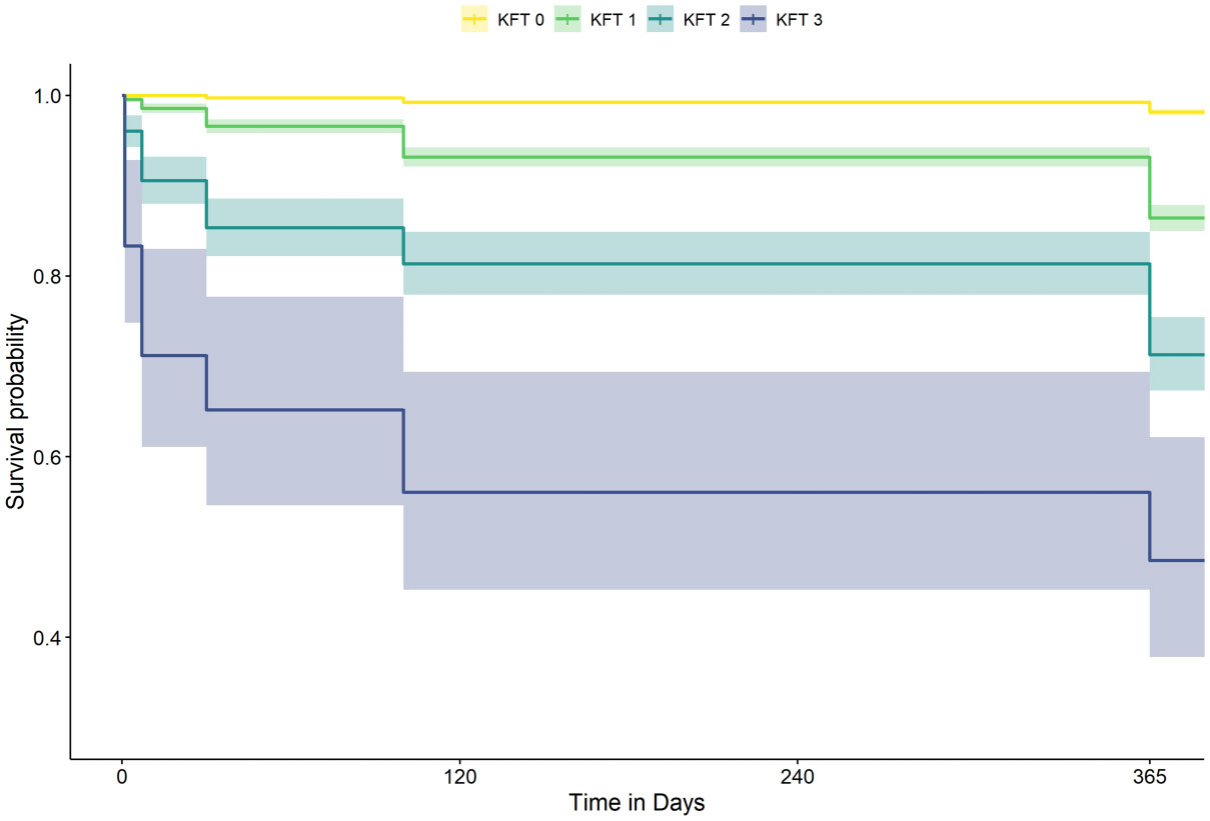
Survival curve of the KFT subgroups for 1-year mortality. To improve readability, the graph was cropped on the *y*-axis (starting at 0.4).

From 30 January 2017 to 20 February 2017 and from 18 March 2019 to 20 May 2019, 11 693 patients presented to our ED. The KFT score could be calculated for 8278 of these patients [mean age 53.7 SD 21.8 years; 3983 (48.1%) female], who thus comprised the final study population (Figure 1); of these patients, 41 (0.5%) died within 24 h, all of whom were admitted. Sixteen (0.19%) patients were lost to follow-up at 7 days and 47 (0.57%) at 1 year. The probability of hospital admission and mortality increased as the KFT score increased (see Tables 1 and 2), and the Kaplan–Meier 1-year survival curves for each of the four KFT score points were all significantly different (Figure 2).

**Table 1.** Baseline demographics and main outcomes divided by KFT

	All	KFT 0	KFT 1	KFT 2	KFT 3
*N* (%)	8278	5544 (66.97)	2189 (26.44)	479 (5.79)	66 (0.80)
Age, mean (SD)	53.74 (21.75)	47.35 (19.25)	65.82 (20.93)	69.97 (19.85)	72.21 (17.36)
Sex, female, *n* (%)	3983 (48.1)	2583 (46.6)	1138 (52.0)	229 (47.8)	33 (50.0)
ESI, *n* (%)					
1	216 (2.6)	10 (0.2)	79 (3.6)	97 (20.3)	30 (45.5)
2	2158 (26.1)	1101 (19.9)	821 (37.5)	217 (45.3)	19 (28.8)
3	3530 (42.6)	2273 (41.0)	1086 (49.6)	155 (32.4)	16 (24.2)
4	2229 (26.9)	2022 (36.5)	197 (9.0)	9 (1.9)	1 (1.5)
5	145 (1.8)	138 (2.5)	6 (0.3)	1 (0.2)	0 (0)
Hospitalization, *n* [missing values] (%)	8278 [0]				
Admission	2971 (35.9)	1151 (20.8)	1382 (63.1)	379 (79.1)	59 (89.4)
Discharge	5307 (64.1)	4393 (79.2)	807 (36.9)	100 (20.9)	7 (10.6)
24-h mortality, *n* [missing values] (%)	8278 [0]				
Dead	41 (0.5)	1 (0.02)	10 (0.5)	19 (4.0)	11 (16.7)
Alive	8237 (99.5)	5543 (99.98)	2179 (99.5)	460 (96.0)	55 (83.3)
7-Day mortality, *n* [missing values] (%)	8262 [16]				
Dead	96 (1.2)	1 (0.02)	31 (1.4)	45 (9.4)	19 (28.8)
Alive	8166 (98.8)	5531 (99.98)	2154 (98.6)	434 (90.6)	47 (71.2)
1-Year mortality, *n* [missing values] (%)	8231 [47]				
Dead	569 (6.9)	102 (1.9)	296 (13.6)	137 (28.7)	34 (51.5)
Alive	7662 (93.1)	5407 (98.1)	1883 (86.4)	340 (71.3)	32 (48.5)

Data are shown as mean and standard deviation for continuous variables and as number and percentage for categorical variables.

**Table 2.** Baseline demographics and main outcomes divided by ESI

	All	ESI 1	ESI 2	ESI 3	ESI 4	ESI 5
*N* (%)	8278	216 (2.60)	2158 (26.07)	3530 (42.64)	2229 (26.92)	145 (1.75)
Age, mean (SD)	53.74 (21.75)	65.50 (18.61)	59.91 (20.32)	56.61 (22.22)	42.73 (18.12)	43.91 (18.84)
Sex, female, *n* (%)	3983 (48.1)	86 (39.8)	1013 (46.9)	1787 (50.6)	1030 (46.2)	67 (46.2)
Hospitalization, *n* [missing values] (%)	8278 [0]					
Admission	2971 (35.9)	202 (93.5)	1283 (59.5)	1381 (39.1)	105 (4.7)	0 (0)
Discharge	5307 (64.1)	14 (6.5)	875 (40.5)	2149 (60.9)	2124 (95.3)	145 (100)
24-h mortality, *n* [missing values] (%)	8278 [0]					
Dead	41 (0.5)	19 (8.8)	13 (0.6)	9 (0.3)	0 (0.0)	0 (0.0)
Alive	8237 (99.5)	197 (91.2)	2145 (99.4)	3521 (99.7)	2229 (100.0)	145 (100.0)
7-Day mortality, *n* [missing values] (%)	8262 [16]					
Dead	96 (1.2)	34 (15.7)	39 (1.8)	23 (0.7)	0 (0.0)	0 (0.0)
Alive	8166 (98.8)	182 (84.3)	2113 (98.2)	3501 (99.4)	2225 (100.0)	145 (100.0)
1-Year mortality, *n* [missing values] (%)	8231 [47]					
Dead	569 (6.9)	63 (29.3)	199 (9.3)	297 (7.9)	27 (2.1)	1 (0.7)
Alive	7662 (93.1)	152 (70.7)	1949 (90.7)	3232 (92.1)	2187 (98.8)	142 (99.3)

Data are shown as mean and standard deviation for continuous variables and as number and percentage for categorical variables. NEWS was only available for 4929 patients.

### Comparison of the KFT score with the ESI

Both triage scores were associated with hospital admission and with both early and late mortality (Tables 1 and 2). The KFT score had a lower discrimination than ESI for hospital admission and had a higher discrimination for mortality from 24 h up to 1 year after presentation (Table 3).

**Table 3.** Area under the curve for 24-h, 7-day and 1-year mortality and admission

	KFT		ESI		*P*-Value
	AUC	95% CI	AUC	95% CI	
24-h mortality	0.92	0.88–0.96	0.84	0.78–0.89	0.005
7-Day mortality	0.92	0.90–0.94	0.82	0.78–0.85	<0.001
1-Year mortality	0.79	0.77–0.81	0.67	0.65–0.69	<0.001
Admission	0.73	0.72–0.73	0.76	0.75–0.77	<0.001

*P*-values were bootstrapped with 100 000 repetitions.

**Figure 3.**
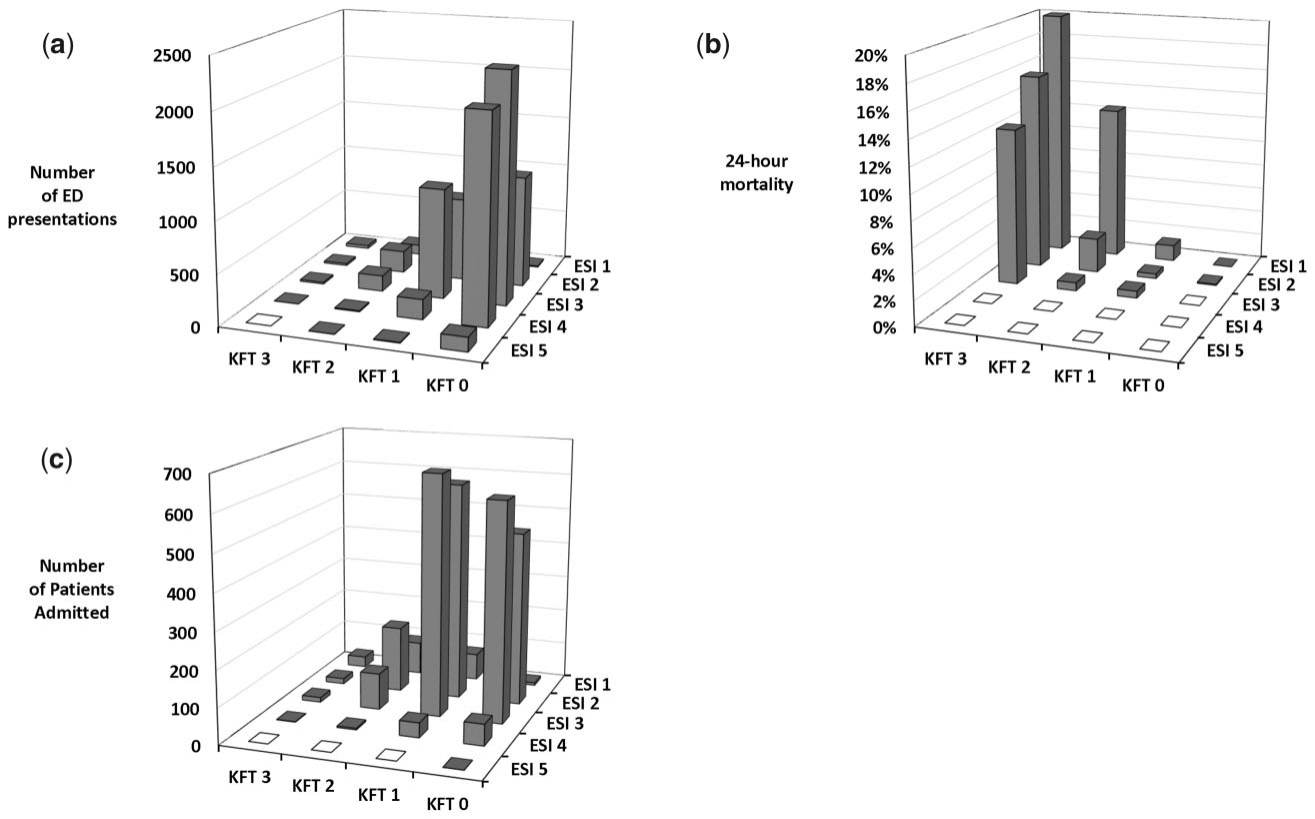
Overview of the distribution of patients between the ESI and the KFT score. (**a**) Number of patients presenting to the ED, divided according to the ESI and the KFT. (**b**) Percentage of ED patients who died within 24 h of presentation, divided according to the ESI and the KFT. (**c**) Number of ED patients who were admitted, divided according to the ESI and the KFT.

There was a significant negative correlation between the KFT score and ESI (*r* = −0.38, *P* < 0.001); of the 8278 ED patients included in the study, 5544 (67.0%) were assigned to the lowest acuity by the KFT score (i.e. zero points) compared with 2374 (28.7%) by the ESI (i.e. levels 4 and 5). Of the 5544 patients with zero KFT points, 10 (0.2%), 1101 (19.9%) and 2273 (41.0%) were assigned to ESI 1, 2 and 3, respectively (Figure 3). Of the 41 patients who died within 24 h, 40 had ≥1 KFT points and none were triaged to ESI 4 or 5 (Figure 3).

The 24-h mortality in patients assigned to low acuity by both scores was very low and not statistically different: 1 (0.02%) of the 5544 patients who scored zero KFT points died within 24 h, whereas none of the 2374 patients assigned to ESI Group 4 and 5 died (*P* = 0.88).

Patients with KFT score >0 had a positive likelihood ratio (+LR) of 2.98 (95% CI: 2.82–3.16) and a negative likelihood ratio (−LR) of 0.04 (95% CI: 0.01–0.25) for 24-h mortality. When comparing KFT score of >1 the +LR is 11.70 (95% CI: 9.55 –14.34) with a −LR of 0.29 (95% CI: 0.17–0.47) for 24-h mortality. Looking at KFT score <3, the +LR is 40.18 (95% CI: 22.72–71.05) with a –LR of 0.74 (95% CI: 0.61–0.89) for 24-h mortality.

More than 60% of patients with KFT 0 were assigned to ESI Group 1–3 (i.e. 3,384 of 5544 patients, 61). Only one patient (0.02%) with KFT 0 died within 24 h. Admission was strongly associated with the ESI; 2866 of the 2971 (96.5%) patients admitted to the hospital had an ESI 1–3, whereas only 1820 (61.3%) of admitted patients had a KFT of ≥1 (OR 3.4, 95% CI 3.1–3.72, *P* < 0.001) (Figure 3).

One patient would have received a KFT score of 0 during triage but died within 24 h. This patient received an ESI score of 2 (should not wait). This mobile nursing home resident was immunocompromised and suffered from sepsis. The patient's condition deteriorated rapidly within 30 min after triage and died within 24 h.

## Discussion

### Main findings

The main results of this study were that the simple and fast KFT triage tool (based on gait, mental status and oxygen saturation) has a lower discrimination for hospital admission, but a higher discrimination for mortality at all time points, as compared to the ESI. The KFT score accurately identifies more than twice as many patients at low risk of 24-h mortality than the ESI.

### Interpretation

ESI strata were more strongly associated with hospital admission than the KFT. Although the ESI is a well-established standard triage process in many hospitals throughout the world, it is constructed around measures of both acuity (e.g. vital parameters) and the prediction of resource consumption. Therefore, it is inextricably linked to the estimated likelihood of hospital admission. While physicians can predict the disposition of ED patients using diagnostic reasoning based on minimal available information,^22^ the decision to admit patients may vary considerably^23^—final disposition being influenced by numerous physician-, patient- and institution-specific factors.^24^ The stringent association between hospital admission and the ESI, which is unrelated to the risk of death predicted by the KFT, may be explained by initial intuitive assessment^25^ driving of both the ESI attribution and the admission decision.

Our findings show that the KFT score can be used as mortality predictor for up to 1 year after ED presentation; this superiority over the ESI regarding prognostic accuracy may support prudent disposition decisions. However, the KFT score does not assess any symptoms, presentations or distress. Certain presentations, even if not life threatening, may require immediate attention to reduce morbidity (e.g. bleeding, stroke-like symptoms and arrhythmias) or suffering (e.g. pain or dyspnoea). In our study, for example, we encountered a patient with sepsis, who deteriorated rapidly and would have not been identified by the KFT. Likewise, hypothetical patients with acute chest pain or hemiparesis might walk into the ED and score 0 points on the KFT but should receive prompt workup. Nevertheless, we think the KFT, if used by a triage clinician, can be useful for the rapid identification of low-risk patients.

In a validation study in Uganda, the KFT score was found to be easy to use, took little time, skills or training and did not significantly increase the workload of clinical staff.^26^ Although it is also our own observation that mental status and mobility can be assessed quickly and easily, the reliability of mental status assessments used by different early warning scores has been questioned,^27^ and the intra- and interobserver variabilities of mobility measures require further investigation.^28^ Moreover, the reliability of the KFT score, defined as interobserver agreement, has not been tested. In contrast, although over-triage and under-triage assessments differ substantially,^11^ expert evaluations of the ESI determined it had >90% accuracy.^29^

In this study, proxy measurements of acuity were short-term mortality and hospital admission. Fully tested triage scores have typically used construct validity regarding mortality, admission, intensive care, length of stay and the use of resources. Although the ESI was designed to assess acuity and predict the use of resources, mortality prediction was not its intended use. Moreover, hospital admission is not the only determinant for resource consumption. To decide on hospitalization, a detailed workup including imaging, clinical chemistry and psycho-social assessments may be needed. It was therefore not our intention to replace the ESI by the KFT, but to assess the construct validities of both tools, so that they might be used together to support triage decisions in times of crowding. It is unclear if deferring the investigation and treatment of patients without vital threat will save resources. Although elective treatment may also be more cost-effective than those performed in the ED, low-risk patients in distress will always require immediate attention that justifies the minimal additional cost of a full investigation.

### Clinical relevance

Advantages of the KFT score are its simplicity (can be performed by anyone without much training), its low demand of time and resources and its reliability and robustness.^30^ Compared to the ESI, the KFT score may identify more patients at low risk of early death.

Triage should not be restricted to reduce short-term mortality but should also include the timely relief of pain and discomfort and reduce acute morbidity. In some emergency systems, such as in the UK, alternative routes to access urgent and emergency care are being implemented. The KFT score may be a useful adjunct in determining which patients may be suitable for streaming to same-day emergency care (SDEC) and urgent treatment centres.

The purpose of this article was to compare KFT with ESI. Comparison with National Early Warning Score (NEWS) and similar early warning scores is difficult because data collection of all the vital signs required to calculate them was often missing. The Triage Early Warning Score, which like NEWS requires a full set of vital signs, has previously been shown to have a comparable performance to KFT.^26^ Measuring a complete set of vital signs at ED triage is a challenge in cases of high urgency, such as with unstable patients, those requiring resuscitation, or those directly referred for surgery. Similarly, in low-urgency cases, triage clinicians may decide to omit the measurement of certain variables. One notable advantage of the KFT is its simplicity as a score that can be feasibly recorded on all individuals.

### Research implications

Future research should try to simplify, accelerate and improve parts of the triage process by automation, IT support and artificial intelligence. Technology would be available to measure vital parameters touch free, to track gait electronically and to offer self-registration potentially assessing mental status or ‘red flag’ complaints.

### Limitations

This study has several limitations. It was a single-centre study performed in the spring and, therefore, may not be applicable to different seasons or to facilities in other parts of the world. Second, as we merged data from 2017 and 2019, the patients were not all consecutive, and >20% of enrolled patients were excluded because of missing data (Figure 1). Third, it is possible that some patients with a normal oxygen saturation were receiving supplemental oxygen. Fourth, only 68 patients had 3 KFT points, compared with 226 assigned to ESI level 1, which may have inflated their odds ratios. Fifth, a walking and talking patient with a good oxygen saturation, which would receive a KFT of 0, might still need to be seen immediately for an urgent presentation (e.g. acute chest pain, hemiparesis). Lastly, the reliability of the KFT to predict imminent mortality may be questionable as there were only 41 deaths within 24 h in our study cohort.

## Conclusion

Compared to the ESI, the KFT score based on mental status, mobility and oxygen saturation identifies more than twice as many ED patients at low risk of early death. Therefore, the KFT score might help identify patients who can safely be managed through alternative pathways such as SDEC. The KFT may be particularly helpful in situations of ED crowding and access block.

## Supplementary Material

hcad160_Supplementary_Data

## References

[1] Weber EJ , McAlpine I, Grimes B. Mandatory triage does not identify high-acuity patients within recommended time frames. Ann Emerg Med 2011; 58:137–42. 21514968 10.1016/j.annemergmed.2011.02.001

[2] Screening questions at triage. Ann Emerg Med 2023; 81:e9. 36543495 10.1016/j.annemergmed.2022.11.006

[3] Johnson KD , Punches BE, Smith CR. Perceptions of the essential components of triage: a qualitative analysis. J Emerg Nurs 2021; 47:192–7. 33097241 10.1016/j.jen.2020.08.009

[4] Jones S , Moulton C, Swift S, Molyneux P, Black S, Mason N, et al Association between delays to patient admission from the emergency department and all-cause 30-day mortality. Emerg Med J 2022; 39:168–73. 35042695 10.1136/emermed-2021-211572

[5] Obermeyer Z , Cohn B, Wilson M, Jena AB, Cutler DM. Early death after discharge from emergency departments: analysis of national US insurance claims data. BMJ 2017; 356:j239. 28148486 10.1136/bmj.j239 PMC6168034

[6] Stanich JA , Oliveira JESL, Ginsburg AD, Mullan AF, Jeffery MM, Bellolio F. Increased short-term mortality among patients presenting with altered mental status to the emergency department: a cohort study. Am J Emerg Med 2022; 51:290–5. 34785485 10.1016/j.ajem.2021.10.034 PMC9376886

[7] Laugesen SKN , Nissen SK, Kellett J, Brabrand M, Cooksley T, Nickel CH. Impaired mobility, rather than frailty, should be a vital sign. Chest 2019; 155:877–8. 30955577 10.1016/j.chest.2018.11.029

[8] Busch JM , Arnold I, Kellett J, Brabrand M, Bingisser R, Nickel CH. Validation of a simple score for mortality prediction in a cohort of unselected emergency patients. Int J Clin Pract 2022; 2022:7281693. 36225535 10.1155/2022/7281693 PMC9525775

[9] Nickel CH , Kellett J, Nieves Ortega R, Lyngholm L, Hanson S, Cooksley T, et al A simple prognostic score predicts one-year mortality of alert and calm emergency department patients: a prospective two-center observational study. Int J Clin Pract 2020; 74:e13481. 31985868 10.1111/ijcp.13481

[10] Kellett J , Sikakulya FK, Nickel CH. The prediction of early mortality by the ROX index of oxygenation and respiratory rate in diverse Canadian and Ugandan cohorts of unselected patient: a post-hoc retrospective analysis of 80,558 patient observations. Acute Med 2022; 21:68–73. 35681179 10.52964/AMJA.0900

[11] Grossmann FF , Bingisser R, Nickel CH. Comment on the validity of emergency department triage tools. Am J Emerg Med 2017; 35:1376. 10.1016/j.ajem.2017.03.054 28366284

[12] Mackway-Jones K , Marsden J, Windle J. Emergency Triage: Manchester Triage Group. Oxford: Wiley, 2013.

[13] Bingisser R , Dietrich M, Nieves Ortega R, Malinovska A, Bosia T, Nickel CH. Systematically assessed symptoms as outcome predictors in emergency patients. Eur J Intern Med 2017; 45:8–12. 29074217 10.1016/j.ejim.2017.09.013

[14] Hudson GR , Howley N, Boyle A. Can artificial intelligence and machine learning help reduce the harms of emergency department crowding? Eur J Emerg Med 2021; 28:95–6. 33290297 10.1097/MEJ.0000000000000781

[15] Pollaris G , Sabbe M. Reverse triage: more than just another method. Eur J Emerg Med 2016; 23:240–7. 26479736 10.1097/MEJ.0000000000000339

[16] De Bondt F , Pollaris G, Sabbe MB. Can a reverse triage clinical decision support tool create sufficient surge capacity and reduce emergency department crowding? Eur J Emerg Med 2022; 29:16–7. 34285173 10.1097/MEJ.0000000000000855

[17] Grossmann FF , Nickel CH, Christ M, Schneider K, Spirig R, Bingisser R. Transporting clinical tools to new settings: cultural adaptation and validation of the Emergency Severity Index in German. Ann Emerg Med 2011; 57:257–64. 20952097 10.1016/j.annemergmed.2010.07.021

[18] Brink A , Alsma J, van Attekum LA, Bramer WM, Zietse R, Lingsma H, et al Predicting inhospital admission at the emergency department: a systematic review. Emerg Med J 2022; 39:191–8. 34711635 10.1136/emermed-2020-210902 PMC8921564

[19] Kikomeko B , Mutiibwa G, Nabatanzi P, Lumala A, Kellett J; Kitovu Hospital Study Group. A prospective, internal validation of an emergency patient triage tool for use in a low resource setting. Afr J Emerg Med 2022; 12:287–92. 35782196 10.1016/j.afjem.2022.05.003 PMC9240986

[20] Hanley JA , McNeil BJ. A method of comparing the areas under receiver operating characteristic curves derived from the same cases. Radiology 1983; 148:839–43. 6878708 10.1148/radiology.148.3.6878708

[21] R Core Team. *R: A Language and Environment for Statistical Computing*. Vienna, Austria, R Foundation for Statistical Computing, 2022. https://www.R-project.org/ (10 July 2023, date last accessed).

[22] Bingisser R , Baerlocher SM, Kuster T, Nieves Ortega R, Nickel CH. Physicians’ disease severity ratings are non-inferior to the Emergency Severity Index. JCM 2020; 9:762. 32168931 10.3390/jcm9030762 PMC7141189

[23] Smulowitz PB , O'Malley AJ, McWilliams JM, Zaborski L, Landon BE. Variation in rates of hospital admission from the emergency department among medicare patients at the regional, hospital, and physician levels. Ann Emerg Med 2021; 78:474–83. 34148659 10.1016/j.annemergmed.2021.03.020

[24] Lewis Hunter AE , Spatz ES, Bernstein SL, Rosenthal MS. Factors influencing hospital admission of non-critically ill patients presenting to the emergency department: a cross-sectional study. J Gen Intern Med 2016; 31:37–44. 26084975 10.1007/s11606-015-3438-8 PMC4700015

[25] Rohacek M , Nickel CH, Dietrich M, Bingisser R. Clinical intuition ratings are associated with morbidity and hospitalisation. Int J Clin Pract 2015; 69:710–7. 25689155 10.1111/ijcp.12606 PMC5024066

[26] Wasingya-Kasereka L , Nabatanzi P, Nakitende I, Nabiryo J, Namujwiga T, Kellett J; Kitovu Hospital Study Group. Two simple replacements for the triage early warning score to facilitate the South African Triage Scale in low resource settings. Afr J Emerg Med 2021; 11:53–9. 33489734 10.1016/j.afjem.2020.11.007 PMC7806646

[27] Brunker C , Harris R. How accurate is the AVPU scale in detecting neurological impairment when used by general ward nurses? An evaluation study using simulation and a questionnaire. Intensive Crit Care Nurs 2015; 31:69–75. 25599998 10.1016/j.iccn.2014.11.003

[28] Sackley C , Richardson P, McDonnell K, Ratib S, Dewey M, Hill HJ. The reliability of balance, mobility and self-care measures in a population of adults with a learning disability known to a physiotherapy service. Clin Rehabil 2005; 19:216–23. 15759538 10.1191/0269215505cr815oa

[29] Gilboy NT , Travers D, Rosenau A. Emergency Severity Index (ESI): A Triage Tool for Emergency Department Care. Rockville, Agency for Healthcare Research and Quality, 2012.

[30] Kellett J. What is the ideal triage process and the resources it requires? Lancet Reg Health West Pac 2021; 13:100203. 34527989 10.1016/j.lanwpc.2021.100203 PMC8403893

