# Supplementary material for: A fast emergency department triage score based on mobility, mental status and oxygen saturation compared with the emergency severity index: a prospective cohort study
Source: QJM. 2023 Jul 3;116(9):774–80. doi: 10.1093/qjmed/hcad160 (PMC10559338; doi:10.1093/qjmed/hcad160)
Supplement: hcad160_Supplementary_Data [file hcad160_supplementary_data.pdf]

Supplemental Appendix A

Table A1: Performance criteria of KFT

The chart displays the performance criteria of the KFT Score when looking at 24-hour mortality. KFT 0 describes the difference of 0 on the KFT score versus 1-3 on the KFT score. KFT 3 describes the difference of 3 on the KFT score versus 0-2 on the KFT score. KFT >1 describes the difference of 2-3 on the KFT score versus 0-1 on the KFT score. KFT, Kitovu Fast Triage Score calculated by oxygen saturation.

	**KFT 0**	**KFT 3**	**KFT >1**
Sensitivity (95% CI)	98% (87% - 100%)	27% (14% - 43%)	73% (57% - 86%)
Specificity (95% CI)	67% (66% - 68%)	99% (99% - 99%)	94% (93% - 94%)
Positive Likelihood Ratio (95% CI)	2.98 (2.82 – 3.16)	40.18 (22.72 – 71.05)	11.70 (9.55 –14.34)
Negative Likelihood Ratio (95% CI)	0.04 (0.01 – 0.25)	0.74 (0.61 – 0.89)	0.29 (0.17 – 0.47)
